# Mid-Borderline Leprosy with Type I Reaction

**DOI:** 10.4269/ajtmh.24-0217

**Published:** 2024-08-13

**Authors:** Jorge C. F. Nakazaki, Malika Madhava, Cesar Ramos

**Affiliations:** ^1^ Instituto de Medicina Tropical “Alexander von Humboldt,” Universidad Peruana Cayetano Heredia, Lima, Peru;; ^2^ Facultad de Medicina Alberto Hurtado, Universidad Peruana Cayetano Heredia, Lima, Peru;; ^3^ William Crawford Gorgas Center for Geographic Medicine, University of Alabama at Birmingham, Birmingham, Alabama;; ^4^ Unidad de Dermatologia, Departamento de Enfermedades Infecciosas, Tropicales y Dermatológicas, Hospital Nacional Cayetano Heredia, Lima, Peru

A 70-year-old male farmer from Jaen, Cajamarca, a region in the high jungle of Peru, presented to the outpatient dermatology clinic with 3 years of nonpainful, erythematous circular plaques. The lesions reportedly started as erythematous, painless patches on the torso and spread over 3 years to the patient’s back and finally to the upper and lower extremities (Figure 1). The patient endorsed dryness in both eyes, numbness in his hands and feet, occasional recent fevers (unquantified), and mild testicular pain. On examination, he was afebrile and the lesions were noted to have scaly, hypochromic centers with hypoesthesia. The exam showed peripheral nerve thickening of both ulnar and posterior tibial nerves and decreased muscle strength and atrophy in the territories of the bilateral ulnar nerves. Corneal reflexes were absent bilaterally.

**Figure 1.**
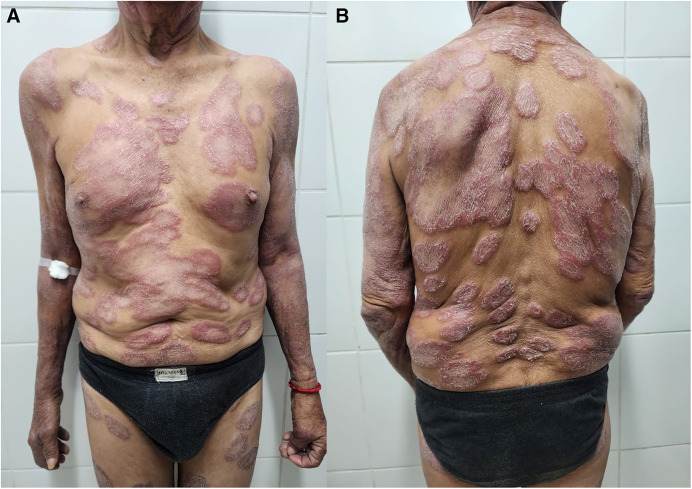
Plaques with erythematous borders and hypochromic, scaly centers are distributed on the patient’s (**A**) torso, upper extremities, and lower extremities, and (**B**) back.

A skin biopsy of one of the abdominal lesions showed sparse *Mycobacterium leprae* on the Fite-Faraco stain, which supports the diagnosis of mid-borderline leprosy (Figure 2). Staining showed moderate lymphohistiocytic inflammatory infiltrate with a linear pattern of non-caseating granulomatous inflammation (Figure 3), which suggests a type 1 reaction. The patient was treated with dapsone, rifampin, and clofazimine per WHO guidelines.^1^

**Figure 2.**
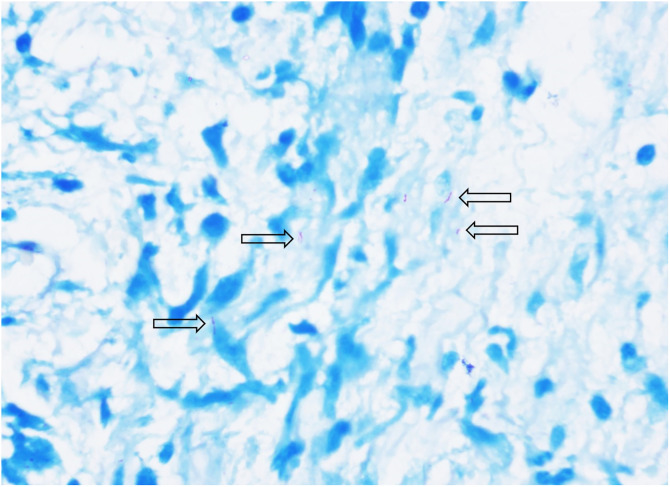
Fite-Faraco stain of abdominal lesion biopsy showing sparse *Mycobacterium leprae*.

**Figure 3.**
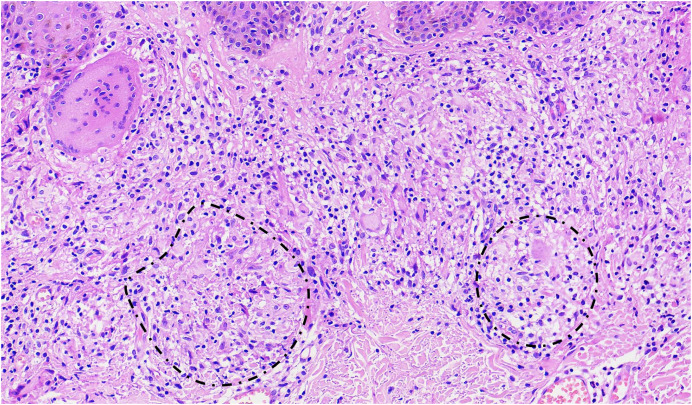
Hematoxylin and eosin stain of an abdominal lesion biopsy shows a moderate lymphohistiocytic inflammatory infiltrate with a linear pattern and the formation of noncaseating epithelioid granulomas (encircled) and multinucleated giant cells.

Leprosy is a bacterial infection caused by *Mycobacterium leprae*, affecting mainly the peripheral nerves and skin. According to the Ridley-Jopling classification, leprosy can present in various clinical forms depending on the host’s immune response against the bacilli.^2^ Our patient presented with multiple asymmetric plaques and “punched-out lesions,” or ring-shaped plaques with a well-defined center and sloping outer edges, classic for mid-borderline leprosy.

Patients with leprosy can present with two significant types of reactions. Type 1 reactions occur owing to a change in cell-mediated immunity. These are most often characterized by erythema and edema of the original lesions, occurring in borderline forms of leprosy or patients starting treatment. Type 2 reactions occur in lepromatous leprosy caused by immune-complex deposition and are most often characterized by erythema nodosum leprosum and systemic signs such as fever and generalized inflammatory response. Type 1 and 2 reactions are treated with anti-inflammatory medications depending on severity.^3^ Our patient’s fever and the erythematous, scaly appearance of his lesions were characteristic of a type 1 reaction; his clinical picture improved rapidly on leprosy treatment alone, with improvement in his lesions after 3 months (Figure 4).

**Figure 4.**
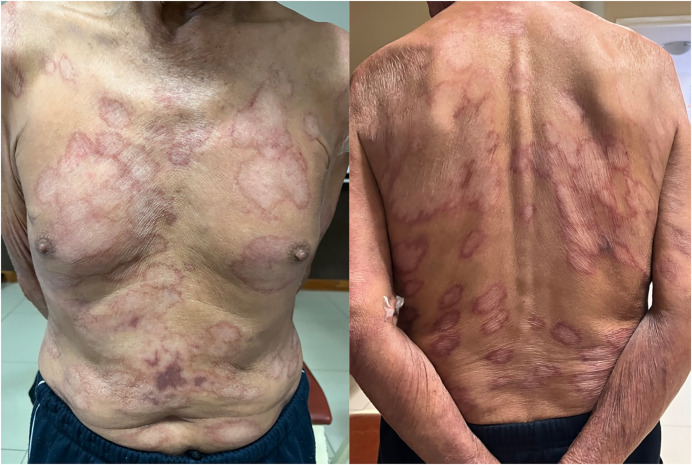
Improvement of lesions after three months of treatment.

## References

[1] World Health Organization, 2018. Guidelines for the Diagnosis, Treatment, and Prevention of Leprosy. Available at: https://apps.who.int/iris/bitstream/handle/10665/274127/9789290226383-eng.pdf?ua=1. Accessed March 31, 2024.

[2] Britton WJ Lockwood DNJ, 2004. Leprosy. Lancet Lond Engl 363: 1209–1219. 10.1016/S0140-6736(04)15952-7 15081655

[3] Bilik L Demir B Cicek D, 2017. Leprosy reactions. Ribón W, ed. Hansen’s Disease – The Forgotten and Neglected Disease. London, United Kingdom: IntechOpen, 81–91.

